# The Complete Mitochondrial Genomes of Two Octopi of the Western Pacific Ocean, *Japetella diaphana* and *Amphitretus pelagicus* (Cephalopoda: Amphitretidae), and Their Phylogenetic Position Within Amphitretidae

**DOI:** 10.3390/genes17030312

**Published:** 2026-03-10

**Authors:** Michel Murwanashyaka, Lihua Jiang, Liyi Pei, Bilin Liu

**Affiliations:** 1 College of Marine Living Resource Sciences and Management, Shanghai Ocean University, Shanghai 201306, China; mikeshyaka@gmail.com (M.M.); liyipei@zjou.edu.cn (L.P.); 2 National Engineering Research Center of Marine Facilities Aquaculture, Zhejiang Ocean University, Zhoushan 316022, China; 3 Department of Sciences, Kibogora Polytechnic, Nyamasheke P.O. Box 50, Rwanda

**Keywords:** *Amphitretus pelagicus*, *Japetella diaphana*, mitogenome, phylogeny

## Abstract

Background/Objectives: A comprehensive analysis of the mitochondrial genomes of *Japetella diaphana* and *Amphitretus pelagicus* was conducted to investigate their genomic composition, gene size, sequence characteristics, and phylogenetic positioning within the Amphitretidae family. Methods: A rigorous phylogenetic analysis was performed utilizing a dataset comprising 13 protein-coding genes, two ribosomal RNAs, and 22 transfer RNAs derived from 26 cephalopod mitochondrial genomes, representing 25 species across seven families, Vampyroteuthidae, Tremoctopodidae, Octopodidae, Enteroctopodidae, Bolitaenidae, Argonautidae, and Amphitretidae, along with outgroup *Nautilus macromphalus*. Results: Notably, both focal species demonstrated a pronounced adenine–thymine bias in their mitochondrial genomes, with *A. pelagicus* exhibiting gene rearrangements and two extensive non-coding regions. The analysis, employing both the maximum likelihood and Bayesian inference methodologies, revealed a monophyletic relationship between Bolitaenidae and Vitreledonellidae, as well as a sister taxon relationship between Amphitretidae and Tremoctopodidae. The majority of species were classified into the Amphitretidae and Bolitaenidae clades, with numerous species exhibiting close phylogenetic relationships. Conclusions: This study provides novel insights into the evolutionary relationships within Octopodiformes, underscoring the significance of mitochondrial genome data in resolving phylogenetic relationships among cephalopods. The findings contribute to our understanding of the evolutionary history of octopi and pose implications for their classification and conservation. Furthermore, the results underscore the necessity for continued research into the evolutionary relationships among cephalopod taxa.

## 1. Introduction

The octopi are classified within the Phylum Mollusca, Class Cephalopoda, Subclass Coleoidea, and order Octopoda, which encompasses two suborders: Cirrata (deep-sea finned octopods) and Incirrata (benthic and pelagic finless octopods). A well-established sister taxon relationship exists between these suborders [1,2,3,4,5], with over 200 species documented globally. The suborder Incirrata is further subdivided into several higher taxonomic groupings, including the superfamily Argonautoidea, which comprises four distinct families of pelagic octopods: Argonautidae, Tremoctopodidae, and Alloposidae, with gelatinous bodies and females that can attain a total length of up to 4 m [6], as well as Ocythoidae now characterized by a unique reproductive strategy involving a hectocotylus, which exhibit biserial arm suckers and pronounced sexual dimorphism, with mature males being significantly smaller than females. In addition to Argonautoidea, Incirrata includes three other pelagic families, Vitreledonellidae, Bolitaenidae, and Amphitretidae, whose members are gelatinous and transparent, and possess uniserial suckers. The radula of Bolitaenidae and Amphitretidae exhibits a distinctive ctenoglossan morphology with multiple cusps per tooth, leading to their historical classification into the separate taxonomic group Ctenoglossa, subordinate to Incirrata [2,7].

In the current taxonomic framework, all remaining incirrate octopods are classified within the diverse family Octopodidae, comprising over 300 predominantly benthic species. Molecular phylogenetic analyses indicate that Octopodidae may be polyphyletic [3,8,9,10,11], although this hypothesis requires further substantiation. Contemporary classification [12,13] divided Octopodidae into four subfamilies (Octopodinae, Bathypolypodinae, Eledoninae, and Graneledoninae) based on ink sac presence and sucker arrangement [14,15]. However, cladistic analyses [16], examining 15 morphological traits across 14 genera, rejected these subfamily classifications due to paraphyletic groupings [13]. Ref. [4] expanded this work to 29 taxa and 66 characters, concluding that subfamily Ctenoglossa is polyphyletic, identifying Bolitaenidae as basal within Incirrata, and dividing the remaining incirrates into two sister clades, one containing Argonautoidea with Vitreledonellidae and Amphitretidae and the other containing Octopodidae, though the internal Octopodidae structure remained unresolved. Ref. [5] similarly provided evidence for monophyletic Octopoda and sister relationships between Cirrata and Incirrata and between Octopoda and Vampyromorpha, corroborating earlier hypotheses [17,18].

The taxonomic classification of Amphitretidae has undergone significant revision, now encompassing three previously recognized families: Bolitaenidae, Amphitretidae, and Vitreledonelidae [14]. The shared ctenoglossan radula provides morphological evidence for their close relationship, while molecular analyses robustly support a monophyletic clade comprising Bolitaeninae and Vitreledonellidae [14]. Family Amphitretidae is represented by a single genus containing Amphitretus pelagicus and Amphitretus thielei [19], characterized by vesicular, transparent, gelatinous bodies and prominent tubular eyes. They inhabit tropical and subtropical marine environments at depths of approximately 1000 to 2000 m or greater, typically near the benthic zone, with a maximum mantle length of 100 mm and a body length up to 300 mm [20]. The Bolitaenidae family encompasses two genera and two recognized species, Bolitaenus pygmaea and Japetella diaphana [21]. Members exhibit heavily pigmented integument, laterally compressed ocular structures, and front arms with single-row wrist suckers. Within Monodiscus, the male’s left third arm is cauline with a swollen lingual lobe. Adult females of Anispecia possess a distinctive ring of oral photophores enabling bioluminescence at depths exceeding 1000 m to attract conspecific males. The maximum recorded mantle length is 200 mm. These organisms are widely distributed across tropical to temperate marine environments, with adults typically inhabiting continental slopes at 1000 to 2500 m, occasionally extending to abyssal plains at 6000 m. Eggs are oviposited at depths of 750–2500 m and primarily develop on the continental shelf [20]. Molecular markers are increasingly recognized as reliable for precise species identification and correct taxonomic placement within genera [22,23,24,25].

The genus Octopus, named after its type species Octopus vulgaris Cuvier, 1797, has recently been redefined as the “Octopus vulgaris species complex” due to molecular phylogenetic studies indicating that this species encompasses multiple distinct species [26,27,28]. According to the World Register of Marine Species (WORMS), the genus Octopus currently includes 73 recognized species [WORMS. http://www.marinespecies.org (accessed online 24 may 2018)]. This elevated species count results from the provisional classification of numerous “octopus-like” taxa with ambiguous taxonomic status [29]. Consequently, Octopus functions as a “catch-all” taxon, creating considerable taxonomic ambiguity and instability, with frequent instances of synonymy and misallocation to incorrect genera [24,30,31]. These ongoing taxonomic revisions highlight the necessity for continued research into phylogenetic relationships among these species. Previous molecular investigations into cephalopod phylogeny have predominantly utilized two mitochondrial genes, cytochrome c oxidase subunit I (COI) and 16S rRNA genes, though these studies yielded limited phylogenetic resolution. Most molecular phylogenetic research within order Octopodiformes has concentrated on narrow ranges of closely related taxa [24,31,32,33,34,35,36,37]. A seminal study by [8] employed partial COI sequences from 28 octopodiform species, providing the first molecular phylogenetic analysis of relationships among and within octopod families, indicating that family Octopodidae is polyphyletic and suggesting that other incirrate families may have diverged from it. Subsequent research by [3,10,11], utilized expanded datasets of three to four mitochondrial genes, parallel to three nuclear genes, reinforcing the polyphyly of family Octopodidae. These studies further posited that families Vitreledonellidae and Bolitaenidae (identified as sister taxa) may have evolved from within Octopodidae via neoteny, whereby pelagic planktonic paralarvae developed into holopelagic taxa. These studies also provided robust support for a sister relationship between Argonautoidea and the remaining incirrate taxa and strong phylogenetic support for a clade of Southern Ocean endemics and deep-sea octopods characterized by uniserial suckers [9,37], utilizing four mitochondrial genes and six nuclear genes of varying coverage, corroborated each of these proposed relationships. In contrast, complete mitochondrial genomes (mitogenomes) have gained prominence as a valuable resource for elucidating the evolutionary history and conducting phylogenetic reconstructions of species, owing to the conservation of the type and number of genes encoded by metazoan mitogenomes [38].

In the present study, we sequenced and analyzed the complete mitochondrial genome (mitogenome) of *Japetella diaphana* and *Amphitretus pelagicus*. We examined the genome composition, the evolutionary rates of protein-coding genes (PCGs), the secondary structure of transfer RNA (tRNA), and the relative synonymous codon usage (RSCU). Furthermore, a phylogenetic analysis was conducted utilizing the complete mitochondrial sequences of other octopus species available in GenBank to ascertain their phylogenetic position within the family Amphitretidae.

## 2. Materials and Methods

### 2.1. Sample Collection and Genomic DNA Extraction

The specimens of *J. diaphana* and *A. pelagicus* were procured via trawling operations in the western Pacific Ocean (coordinates: 36°41′ N, 157°39′ W) in October 2021. All specimens utilized in this study were collected and handled in a manner consistent with ethical guidelines, and this research adheres to the principles outlined in the ARRIVE guidelines. Fresh muscle tissues from these specimens were meticulously dissected and subsequently preserved in 95% ethanol prior to the extraction of genomic DNA. DNA extraction was conducted utilizing the rapid salting-out method [39]. The integrity and quality of the extracted DNA were evaluated by employing 1% agarose gel electrophoresis, and the DNA was then stored at −20 °C for future analyses.

### 2.2. Library Construction, Mitogenome Assembly, and Annotation

The complete mitogenomes of *J. diaphana* and *A. pelagicus* were sequenced utilizing Illumina HiSeq and PacBio sequencing technologies, conducted by Origin Gene Bio-pharm Technology Co., Ltd. (Shanghai, China). DNA libraries were prepared and sequenced using the TruSeq™ Nano DNA Sample Prep Kit (San Diego, CA, USA). Following library preparation, enrichment was performed via a polymerase chain reaction (PCR) using the cBot TruSeq PE Cluster Kit v3-cBot-HS platform (San Diego, CA, USA), after which paired-end sequencing was executed utilizing the TruSeq SBS array (300 cycles). A total of 1421.7 Mb of raw sequencing data was generated. The raw data were subsequently processed using Trimmomatic-0.39 [40] to yield 1329 Mb of clean data. The Q20 quality score of the clean data was determined to be 96.91%. Additionally, the genomic DNA was fragmented into 10 kb segments using the G-tube method [41]. A single-stranded circular library was annealed and ligated with polymerase at the base of a fixed zero-mode waveguide (ZMW) aperture. Sequencing was conducted subsequent to the completion of the assembly process utilizing the PacBio Sequel platform. The N50 value of the filtered PacBio sub-sequences was determined to be 3870 bp. The splice clean data were processed using SPAdes v3.10.1 [42]. Sequences exhibiting adequate coverage and extended assembly length were designated as candidate sequences and compared against entries in the National Center for Biotechnology Information database (http://www.ncbi.nlm.nih.gov/ (accessed on 24 March 2022)) to validate mitochondrial reference sequences. GapCloser v1.12 [43] was employed to align clean reads with the assembled frames, thereby facilitating the filling of local gaps and optimizing the assembly results for paired-end reads and overlaps. The complete mitochondrial genome of Illex argentinus [44] served as a reference genome to correct the starting point and orientation of the mitochondrial assembly sequence, leading to the finalization of the mitochondrial genome sequence.

### 2.3. Sequence Alignments

Mitos software 2.1.7 (http://mitos.bioinf.uni-leipzig.de/index.py (accessed on 6 March 2024)) was employed to predict protein-coding genes (PCGs), transfer RNA (tRNA), and ribosomal RNA (rRNA) genes within the mitochondrial genome [45]. Redundant gene sequences were eliminated, and the positions of start and stop codons were manually adjusted to ensure a set of conserved genes with enhanced accuracy. The verification of start and stop codons was conducted by referencing the previously published mitochondrial genome of octopods [43,44]. The circular features of the mitochondrial genomes of *J. diaphana* and *A. pelagicus* were visualized utilizing the online software CGView version 2 (http://stothard.afns.ualberta.ca/cgview_server/ (accessed on 26 July 2023)) [46]. Relative synonymous codon usage (RSCU) analysis was performed using MEGA X software [47]. Component slope values were calculated using the formulas established by [48]: AT slope = (A − T)/(A + T); GC slope = (G − C)/(G + C). Phylogenetic relationships were reconstructed based on 13 PCGs, 2 rRNAs, and 22 tRNAs derived from 26 cephalopod mitochondrial genomes, including the newly sequenced J. diaphana and A. pelagicus, with Nautilus macromphalus [49] serving as an outgroup (Table 1). Subsequent to manual corrections, the sequenced mitochondrial genomes were submitted to the GenBank database at the National Center for Biotechnology Information (NCBI) to secure GenBank accession numbers (http://www.ncbi.nlm.nih.gov/GenBank/ (accessed on 24 March 2022)).

### 2.4. Phylogenetic Analysis

The substitution saturation of 13 protein-coding genes (PCGs) within the mitochondrial genome of 26 octopus species was quantified utilizing DAMBE7 software [73]. Based on these findings, we aligned the nucleotide sequences of the 13 PCGs according to default parameters in MEGA X [46] to construct maximum likelihood (ML) and Bayesian inference (BI) phylogenetic trees. The complete mitochondrial genomes of the 26 cephalopod species (Table 1) provided the sequence data for the phylogenetic analysis. The phylogenetic relationships were assessed using the ML method, with trees constructed in IQ-TREE [74] employing the optimal “GTR + F + R7” model, supported by 1000 nonparametric bootstrapping replicates. The most suitable ML model was determined using the ModelFinder software version 1.4.2 results [74,75]. The optimal model (GTR + I + G) for each segment was identified through the Akaike Information Criterion (AIC) in MrModeltest 2.3 [76], although this approach raises questions regarding the rationale behind incorporating invariant positions in conjunction with the gamma distribution, particularly given the potential interdependence of parameter estimations [77]. Subsequently, BI analysis was conducted using MrBayes 3.2, in conjunction with PAUP 4.0 [78] and Modeltest 3.7 software within the MrMTgui interface [79]. The BI analyses employed Markov Chain Monte Carlo (MCMC) methods with default settings across three independent runs for 2,000,000 generations, with sampling occurring every 1000 steps. The average standard deviation of split frequencies was maintained at <0.01, and the initial 25% of samples were discarded as burn-in. The resulting phylogenetic tree was visualized using FigTree v1.4.3 (https://github.com/rambaut/figtree/) (accessed on 26 July 2023) [80].

## 3. Results

### 3.1. The General Characteristics of the Mitochondrial Genome

**Figure 1:**
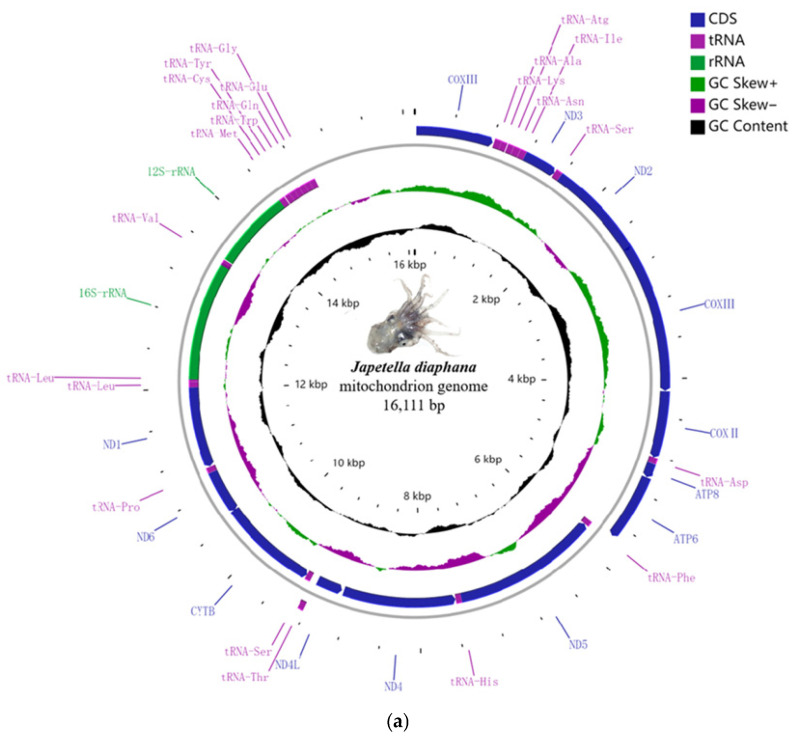

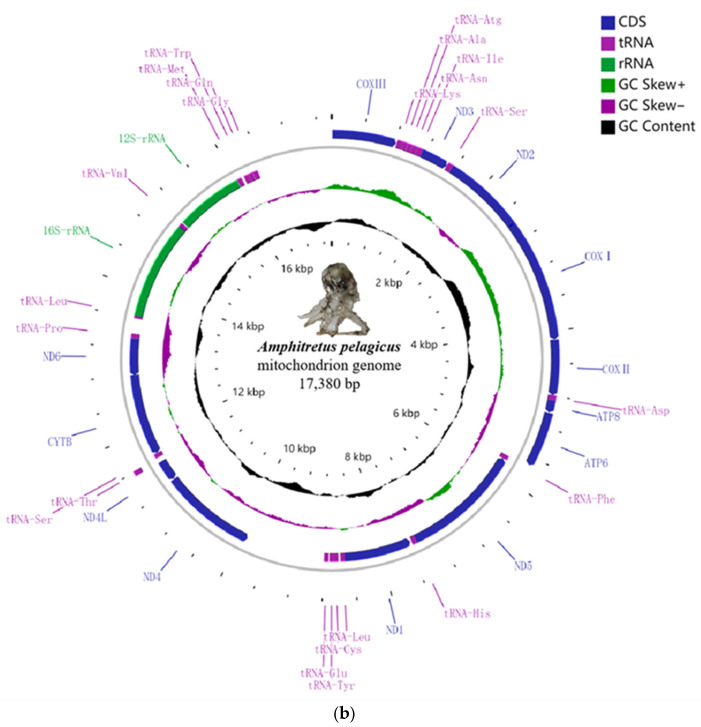
(**a**) Mitochondrial genome map of *J. diaphana*: The two inner circles illustrate the total GC content (in black) and the GC skew (green indicating positive and purple indicating negative) of the mitochondrial genome. The next circles represent the nucleotide identity between *J. diaphana* and other species within the genus *Japetella*. The outermost sections represent the various elements: protein-coding genes (in blue), tRNAs (in purple), rRNAs (in green), and the control region (represented by empty space). Genes with a clockwise arrow are encoded on the “L” strand, while those with a counterclockwise arrow are encoded on the “H” strand. (**b**) Mitochondrial genome map of *A. pelagicus*: The two inner circles illustrate the total GC content (in black) and the GC skew (green indicating positive and purple indicating negative) of the mitochondrial genome. The next circles represent the nucleotide similarity between *A. pelagicus* and other species within the genus *Amphitretus*. The outermost sections represent the various gene types: protein-coding genes (in blue), tRNAs (in purple), rRNAs (in green), and the control region (represented by empty space). Genes with a clockwise arrow are encoded on the “L” strand, while those with a counterclockwise arrow are encoded on the “H” strand.

We accomplished the complete sequencing of the mitochondrial genome of *Japetella diaphana* and *Amphitretus pelagicus* for the first time, with accession numbers ON060363 and ON060364, respectively. The lengths of the mitochondrial genomes were 16,111 base pairs (bp) on GenBank for *J. diaphana* and 17,380 bp for *A. pelagicus* (see Figure 1a,b). Both mitochondrial genomes exhibited the canonical repertoire of 13 protein-coding genes (PCGs), which include cytochrome c oxidase subunits COI-COIII, ATP synthase subunits ATP6 and ATP8, and NADH dehydrogenase subunits ND1-ND6, as well as ND4L. Additionally, each genome contained 22 tRNA genes and 2 rRNA genes (12S and 16S rRNA) (Figure 1a,b). A notable difference between the two species was the presence of a single long non-coding region (LNCR) in *J. diaphana*, while *A. pelagicus* had two such regions (Table 2 and Table 3). The LNCR in *J. diaphana* was located between the trnE and COIII genes, whereas in *A. pelagicus*, the LNCRs were positioned between trnE and ND4 and between trnG and COIII.

The overall base composition of *J. diaphana* was 40.6% adenine (A), 36.3% thymine (T), 15.4% cytosine (C), and 7.7% guanine (G), resulting in an A + T content of 76.9% and a C + G content of 23.1%. This mitochondrial genome exhibited a pronounced AT bias, consistent with the patterns observed in most Cephalopoda species (see Table 2). Furthermore, the entire mitogenome manifested a negative GC skew of −0.333 and a positive AT skew of 0.056. In contrast, the base composition of *A. pelagicus* differed with 40.6% A, 35.1% T, 16.8% C, and 7.5% G, leading to an A + T content of 75.7% and a C + G content of 24.3%. This mitochondrial genome similarly exhibited a significant AT bias, aligning with the trends identified in the majority of Cephalopoda species (refer to Table 2). The overall mitochondrial genome manifested a negative GC skew of −0.383 and a positive AT skew of 0.073.

Both mitochondrial genomes exhibited a composition of 13 protein-coding genes (PCGs), 22 transfer RNA genes (tRNA), 2 ribosomal RNA genes (rRNA), and a single control region (CR). The arrangement of these genes within the mitochondrial genome is consistent with that observed in other previously documented octopus species (Figure 1a,b; Table 2 and Table 3). Specifically, seven PCGs and eight tRNA genes are situated on the light strand (L), whereas the remaining PCGs, the majority of tRNA genes (excluding tRNA-T), and the two ribosomal RNAs are localized on the heavy strand (H) (Table 2 and Table 3). The positioning of these genes on each strand was ascertained based on the guanine and cytosine content, with the L-strand exhibiting a higher cytosine content than the guanine-rich H-strand.

### 3.2. Protein-Coding Genes

The length of the protein-coding genes (PCGs) in *J. diaphana* ranged from 351 bp to 1693 bp. The codon usage for the 13 protein-coding genes is presented in Table 2. The findings revealed that COIII initiates with the codon ATT, while NADH dehydrogenase subunit 4 (ND4) commences with ATA; the remaining PCGs all initiate with ATG. The stop codons employed include TAA for the gene pairs COI-COIII, ATP8, ND1-ND4, and Cytb and TAG for ATP6, ND4L, and ND6, while ND5 exhibits an incomplete stop codon of T.

**Figure 2:**
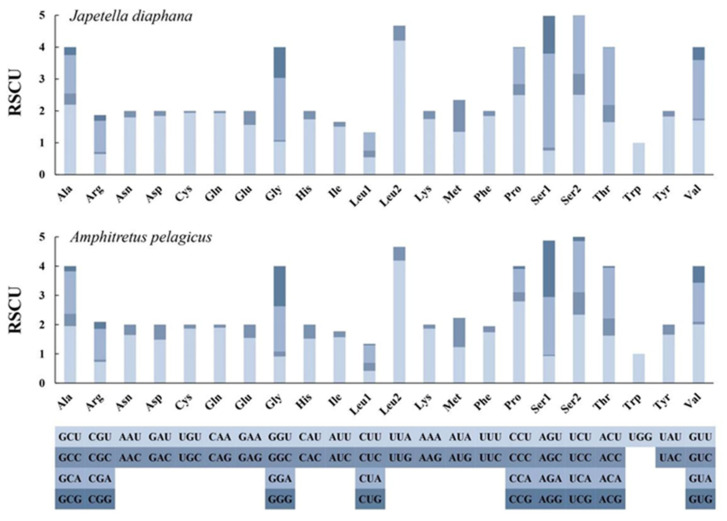
The relative synonymous codon usage of 13 protein-coding genes in the mitogenome for *J. diaphana* and *A. pelagicus*.

In *A. pelagicus*, the lengths of protein-coding genes (PCGs) varied from 351 bp to 1693 bp, with the codon usage of the 13 protein-coding genes detailed in Table 3. Notably, with the exceptions of COIII, ATP6, ND1, and ND4, which utilize ATA and ATT as start codons, respectively, all other PCGs initiate with the codon ATG. Additionally, the stop codons employed include TAA for the gene clusters COI-III, ATP8, ND2, ND3, and Cytb, while TAG is utilized by ATP6, ND1, ND4, ND4L, and ND6; ND5 exhibits an incomplete stop codon of T. The relative synonymous codon usage (RSCU) analysis for the 13 PCGs across these two species suggests a conserved preference for synonymous codons (Figure 2). The most frequently utilized codon in both species is TTA, coding for leucine (Leu2).

**Figure 3:**
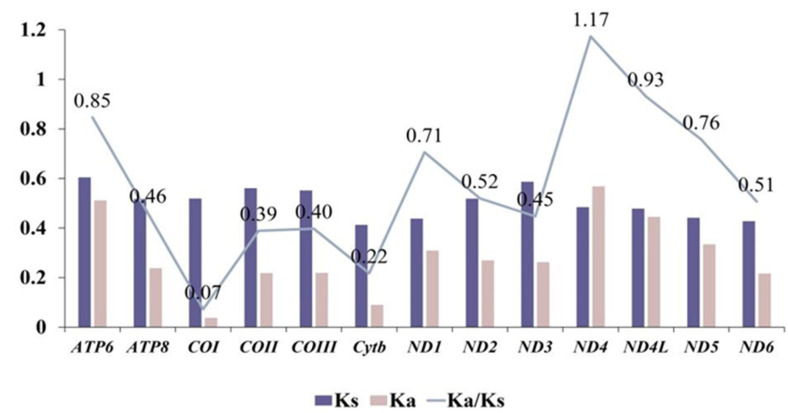
An analysis of protein-coding genes based on the Ka/Ks ratio.

The ratios of nonsynonymous to synonymous substitution rates (Ka/Ks) for the 13 PCGs were calculated using data from 25 Octopodiform species (Figure 3). The Ka/Ks analysis revealed values ranging from 0.07 for cytochrome c oxidase subunit I (COI) to 1.17 for NADH dehydrogenase subunit 4 (ND4). The majority of genes exhibited Ka/Ks values of less than 1, indicative of purifying selection. In contrast, ND4 demonstrated a Ka/Ks value of 1.17, suggesting that it is subject to positive selection. Among the 13 protein-coding genes (PCGs), COI displayed the lowest and most conserved Ka/Ks value, supporting its applicability as a widely utilized DNA barcode for cephalopod identification [81,82].

### 3.3. Transfer and Ribosomal RNAs

**Figure 4:**
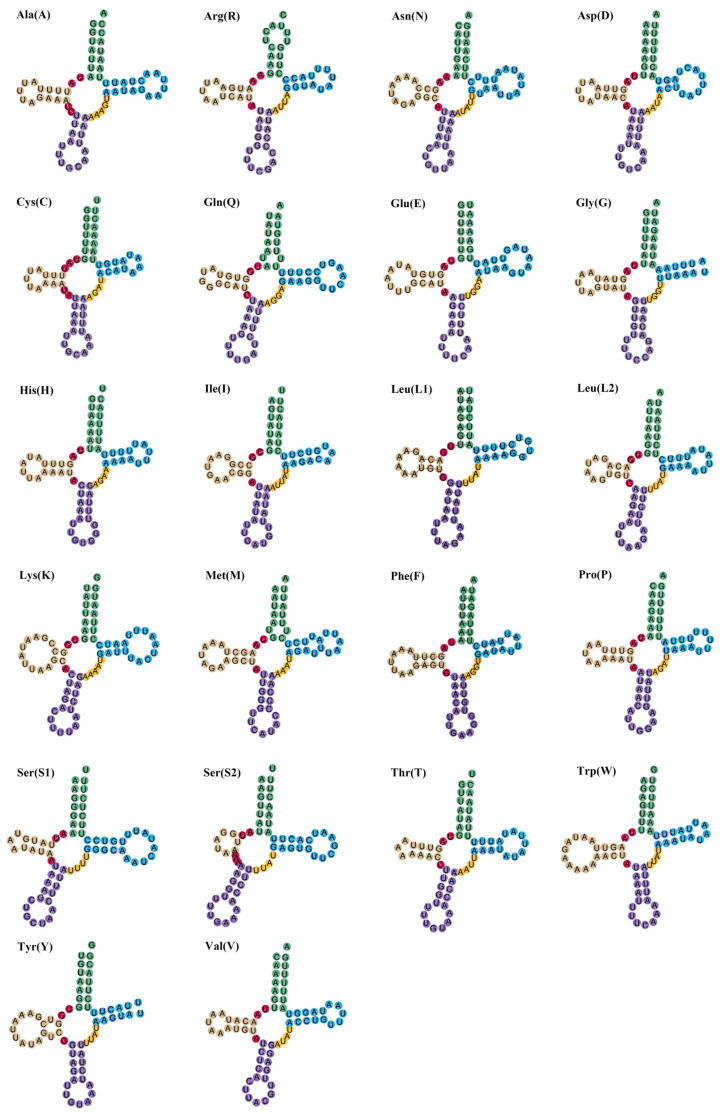
Transfer RNA structure of *J. diaphana*.

**Figure 5:**
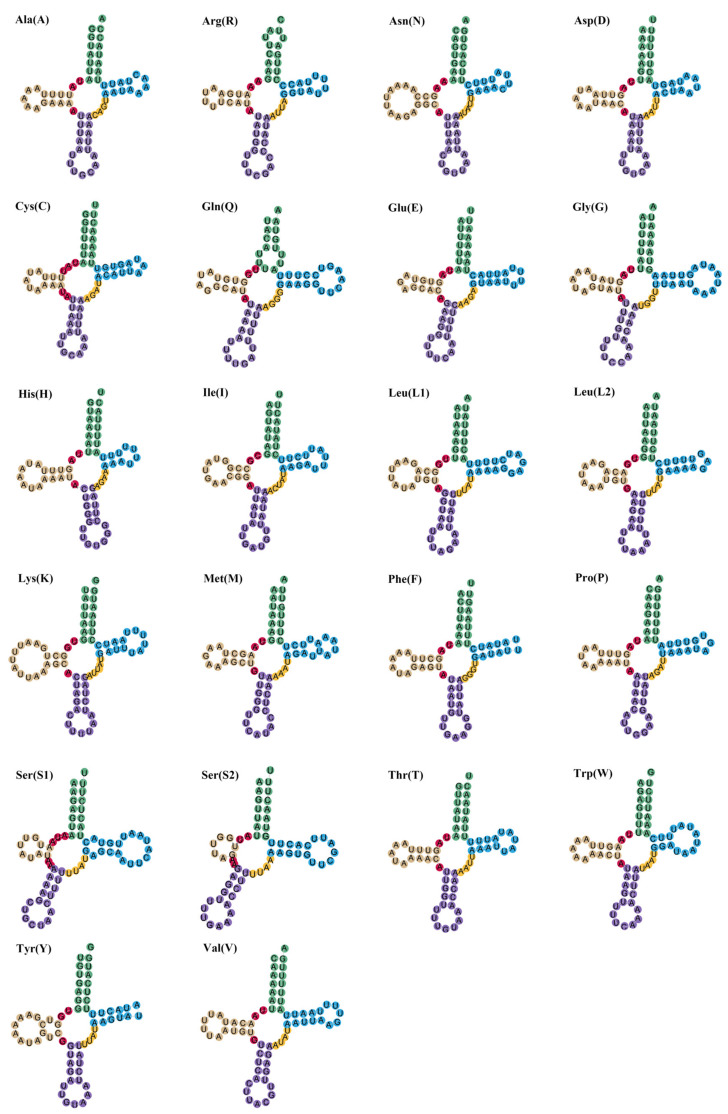
Transfer RNA structure of *A. pelagicus*.

The mitochondrial genomes of the two investigated species encompass a total of 22 transfer RNA (tRNA) genes. In *J. diaphana*, the tRNA genes measure 1462 bp in length, with individual tRNA lengths varying from 63 to 71 bp. Collectively, in *A. pelagicus*, the total length of the tRNA genes is 1448 bp, with lengths ranging from 62 to 70 bp (Table 2 and Table 3). Both species exhibited a negative adenine–thymine (A-T) skew alongside a positive guanine–cytosine (G-C) skew (Table 4). The predicted secondary structures of the tRNA cloverleaf configurations for both species are illustrated in Figure 4 and Figure 5. In *J. diaphana*, the majority of tRNAs exhibited a canonical cloverleaf structure; however, the tRNA for serine (trnSer2) is notable for the absence of the dihydrouridine (DHU) arm, while the TΨC loop is lacking in both trnY and trnG. In the case of *A. pelagicus*, the absence of the DHU arm was observed in both trnS2 and trnS1, a phenomenon frequently documented in metazoans [83,84]. Furthermore, the transfer RNAs for phenylalanine (trnF) and tyrosine (trnY) also lack the TΨC loop.

In the mitochondrial genome of *J. diaphana*, the lengths of the 12S and 16S ribosomal RNA (rRNA) genes were 915 bp and 1397 bp, respectively. In contrast, *A. pelagicus* exhibited lengths of 946 bp for the 12S rRNA and 1398 bp for the 16S rRNA. Notably, in both species, the 12S and 16S rRNA genes were interspersed by the transfer RNA gene (trnV). Furthermore, the ribosomal RNA sequences in these species displayed a negative adenine–thymine (A-T) skew and a positive guanine–cytosine (G-C) skew (Table 4).

### 3.4. Gene Rearrangements

**Figure 6:**
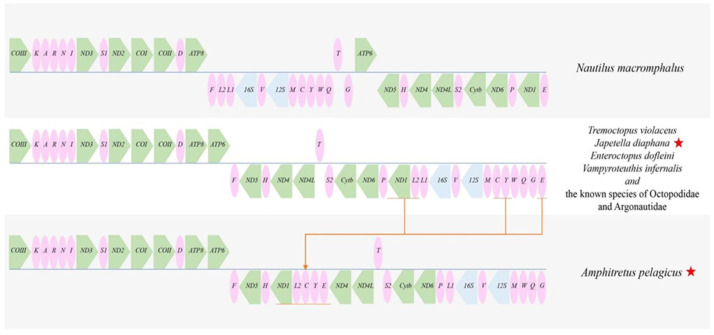
A linearized representation of the mitochondrial gene arrangement. The two species whose sequences are newly reported here are indicated with a red star.

To visualize genomic rearrangements, mitochondrial genes were linearized. Figure 6 depicts the linearization of the protein-coding genes (PCGs), along with the 12S and 16S ribosomal RNA (rRNA) genes across seven families. Despite considerable sequence variation, a conserved gene block was identified. Specifically, three genes (ND3, COI, and COIII), in addition to the ND4-ND4L gene block, were conserved across both focal species. Further, two transfer RNA (tRNA) blocks, designated KARNI and MCYWQG, are illustrated in Figure 6. The KARNI block is located between COIII and ND3, whereas the MCYWQG blocks are situated adjacent to the 12S rRNA gene. Notably, both tRNA blocks underwent rearrangement within family Amphithretidae. The tRNA arrangement observed in *Vampyroteuthis infernalis* is consistent with that of the majority of octopods. All species examined, with the exception of *A. pelagicus*, displayed the L1-L2 gene block positioned adjacent to NADH dehydrogenase subunit 1 (ND1).

For all cephalopods species with known mitochondrial genomes, genomic rearrangements are predominantly observed within the order Decapodiformes. While the gene segments encompassing COIII-trnK-trnA-trnR-trnN-trnI-ND3-trnS1-ND2-COI-COII-trnD-ATP8-trnF-ND5-trnH remain conserved across other Octopodiform species, *A. pelagicus* displays the insertion of ND1-trnL2 and trnC-trnY-trnE between trnH and ND4. Furthermore, *A. pelagicus* contains two long non-coding regions (LNCRs): one located between COIII and trnG and another situated between trnE and ND4. This observed rearrangement may be attributed to random duplication and subsequent loss, as suggested by [85], which could explain the gene rearrangement within this lineage.

### 3.5. Phylogenetic Analysis

**Figure 7:**
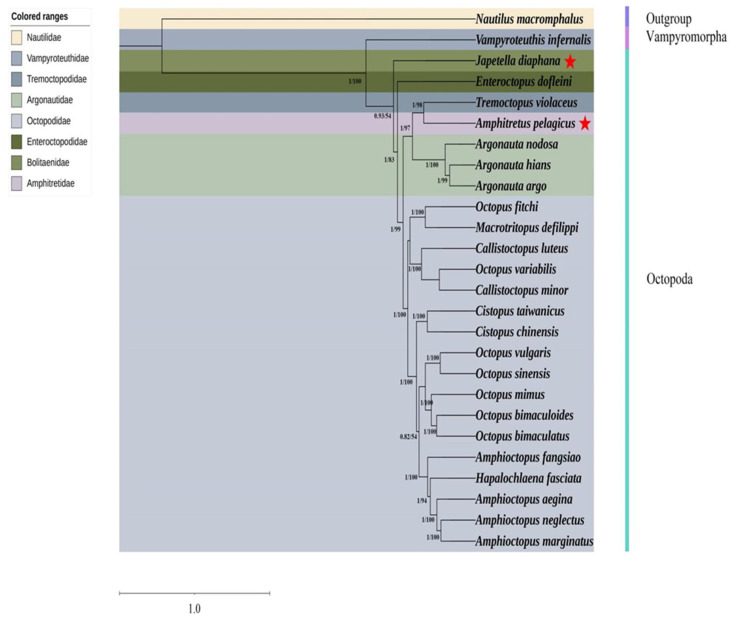
A phylogenetic tree of 26 cephalopod species constructed by the Bayesian inference and maximum likelihood methods based on the concatenated sequences of 13 PCGs and two rRNAs. The two species whose sequences are newly reported here are indicated with a red star.

The mitochondrial genomes of 25 species representing seven families (Vampyroteuthidae, Tremoctopodidae, Argonautidae, Octopodidae, Enteroctopodidae, Bolitaenidae, and Amphitretidae), which are really Octopodiformes but, as Vampyromorpha, distinct from the order Octopoda, thus may serve as an outgroup, especially *Nautilus macromphalus*, which was utilized to construct a phylogenetic tree (Table 1, Figure 7). Both the maximum likelihood (ML) and Bayesian inference (BI) methodologies yielded identical topological structures, exhibiting high support for the majority of branches and categorizing the 26 species into eight primary clades. *N. macromphalus* emerged as a distinct branch. The inferred phylogenetic relationships among the families within Octopodiformes are as follows: ((((((Octopodidae, Argonautidae), Amphitretidae), Tremoctopodidae), Enteroctopodidae), Bolitaenidae), Vampyroteuthidae). Among the sequenced species, *E. dofleini* and *J. diaphana* were found to be closely related. Additionally, *T. violaceus* was identified as a sister group to *A. pelagicus*. Notably, family Octopodidae encompasses the majority of species within order Octopoda; thus the nominal genera are totally mixed within Octopodidae, which strongly supports the revision of the genus-level taxonomy of Octopodidae.

## 4. Discussion

The scarcity of data presents considerable challenges for the investigation of *J. diaphana* and *A. pelagicus*. These species have garnered less research attention compared to other cephalopods, despite their ecological significance and unique morphological characteristics. Their primary habitats, predominantly located in the deep seas of the western Pacific Ocean, remain under-sampled, hindering a comprehensive understanding of their biology, behavior, and ecological roles. Moreover, the reconstruction of their evolutionary histories and phylogenetic relationships within family Amphitretidae is impeded by the absence of sufficient genetic and genomic data. Mitochondrial gene sequences in invertebrates exhibit gene rearrangements on varying scales. In our study, we compared the mitochondrial genomes of currently sequenced species within the order Octopodiformes to the ancestral lineages represented by *N. macromphalus* (Figure 6). Our analysis revealed that the mitochondrial gene order of most species was conserved; however, *A. pelagicus* demonstrated significant translocation and random duplication–loss events. Notably, the genetic sequences of *A. pelagicus* were markedly altered to those of *N. macromphalus*.

The Ka/Ks ratio is an essential metric for evaluating the direction and intensity of natural selection exerted on protein-coding genes. A ratio exceeding 1 indicates the presence of positive or Darwinian selection, which facilitates evolutionary change [86]. In contrast, a ratio below 1 suggests the action of purifying or stabilizing selection, which counteracts evolutionary alterations, while a ratio equal to 1 denotes neutrality, indicating the absence of selective pressure. However, the simultaneous influence of both positive and purifying selection at various loci within a gene or across different temporal stages of its evolution may obscure the detection of one type of selection or affect the apparent strength of another. In our study, the majority of examined genes exhibited Ka/Ks ratios below 1, indicating their susceptibility to purifying selection. However, the ND4 gene displayed a ratio of 1.17, surpassing the threshold of 1, attributable to instances of positive selection during evolutionary history [87]. The conserved usage of synonymous codons, alongside the observed patterns of purifying and positive selection within protein-coding genes, provides a nuanced understanding of the evolutionary pressures influencing these genomes. Among the 13 PCGs, COI is recognized as the most conserved and smallest, frequently employed as a DNA barcode for the identification of cephalopods. The taxonomic classification of octopi has been contentious, with phylogenetic analyses yielding divergent results depending on the mitochondrial genes utilized [81]. Mitochondrial DNA sequences have offered compelling insights into the phylogenetic relationships among octopus species; phylogenetic analysis has corroborated the monophyletic relationship between families Bolitaenidae and Vitreledonellidae [13]. This result underscores the pivotal role of mitochondrial genome data in elucidating the evolutionary relationships among cephalopod species.

This study provides and analyzes the first complete mitochondrial genome sequences for *J. diaphana* and *A. pelagicus*, contributing novel insights into the mitochondrial genomic characteristics, gene arrangement, and phylogenetic relationships within the Octopodiformes clade. Bayesian probabilities and bootstrap supports are provided for each clade, enhancing the robustness of the phylogenetic interpretations. The pronounced AT bias identified in both species corroborates the findings from prior research on cephalopod mitochondrial genomes, underscoring a prevalent trend within this taxonomic group. Notably, *A. pelagicus* harbors two long non-coding regions (LNCRs), and the observed gene rearrangements exhibit significant deviations from the canonical mitochondrial genome organization documented in cephalopods. These results imply that the mechanisms of gene duplication and random loss may be pivotal in shaping the evolutionary trajectory of the mitochondrial genome in this particular species.

## 5. Conclusions

The complete mitochondrial genomes of two octopus species, *J. diaphana* and *A. pelagicus*, were sequenced and analyzed to ascertain their phylogenetic positioning within family Amphitretidae. The results indicated a significant AT bias in the mitochondrial genomes of both species, with *A. pelagicus* exhibiting a notable gene rearrangement and two long non-coding regions (LNCRs). Phylogenetic analysis, employing concatenated sequences from 13 protein-coding genes and two rRNA genes, revealed a monophyletic relationship between families Bolitaenidae and Vitreledonellidae, as well as a sister taxon relationship between *Tremoctopus violaceus* and *Amphitretus pelagicus*. This study provided novel insights into the evolutionary relationships within order Octopodiformes and underscored the utility of mitochondrial genomic data in elucidating phylogenetic relationships among cephalopods. The findings contribute to a deeper understanding of the evolutionary history of octopi and pose significant implications for the conservation of these species.

## Figures and Tables

**Table 1:** List of mitogenomes analyzed in this study. The two species whose sequences are newly reported here are indicated with a star.

Family	Species	Size (bp)	Accession No.	Reference
Amphitretidae	*Amphitretus pelagicus* *	17,380	ON060364	[50] Unpublished
Argonautidae	*Argonauta argo*	15,741	LC596061	[51]
	*Argonauta hians*	16,130	NC036354	[52]
	*Argonauta nodosa*	15,201	NC048475	[53] Unpublished
Bolitaenidae	*Japetella diaphana* *	16,111	ON060363	[50] Unpublished
Enteroctopodidae	*Enteroctopus dofleini*	16,026	NC056385	[54] Unpublished
Octopodidae	*Amphioctopus aegina*	15,545	NC029702	[55]
	*Amphioctopus fangsiao*	15,979	AB240156	[56]
	*Amphioctopus marginatus*	15,719	NC036351	[57]
	*Amphioctopus neglectus*	15,814	MH899749	[58]
	*Cistopus taiwanicus*	15,793	NC023257	[59]
	*Callistoctopus luteus*	15,961	NC039848	[60] Unpublished
	*Cistopus chinensis*	15,706	KF017606	[59]
	*Callistoctopus minor*	15,974	NC015896	[61]
	*Hapalochlaena fasciata*	15,479	NC051545	[62] Unpublished
	*Macrotritopus defilippi*	15,501	MZ264859	[63]
	*Octopus bimaculatus*	16,084	NC028547	[64]
	*Octopus bimaculoides*	15,733	NC029723	[65] Unpublished
	*Octopus fitchi*	15,780	MK450541	[66]
	*Octopus mimus*	15,696	NC044093	[66]
	*Octopus variabilis*	15,992	MF029677	[67] Unpublished
	*Octopus vulgaris*	15,744	AB158363	[68]
	*Octopus sinensis*	15,737	MT712046	[69]
Tremoctopodidae	*Tremoctopus violaceus*	16,015	KY649286	[70]
Vampyroteuthidae	*Vampyroteuthis infernalis*	15,617	NC009689	[71]
Nautilidae	*Nautilus macromphalus*	16,258	DQ472026	[72]

**Table 2:** Gene annotations for the complete mitogenomes of *J. diaphana*.

Gene	Position		Size (bp)		Codon		Intergenic Nucleotides	Strand
	From	To	Nucleotide	Amino Acid	Initiation	Stop		
*COIII*	17	808	792	263	ATT	TAA	0	L
*trnK*	820	888	69				0	L
*trnA*	889	956	68				0	L
*trnR*	965	1027	63				0	L
*trnN*	1029	1098	70				23	L
*trnI*	1100	1165	66				4	L
*ND3*	1166	1516	351	116	ATG	TAA	11	L
*trnS1*	1515	1583	69				9	L
*ND2*	1584	2621	1038	345	ATG	TAA	0	L
*COI*	2593	4125	1533	510	ATG	TAA	8	L
*COII*	4129	4818	690	229	ATG	TAA	1	L
*trnD*	4817	4882	66				−2	L
*ATP8*	4884	5036	153	51	ATG	TAA	1	L
*ATP6*	5039	5731	693	230	ATG	TAG	0	L
*trnF*	5755	5820	66				−8	H
*ND5*	5821	7513	1693	563	ATG	T--	−2	H
*trnH*	7514	7579	66				1	H
*ND4*	7580	8896	1317	438	ATA	TAA	0	H
*ND4L*	8920	9216	297	98	ATG	TAG	−29	H
*trnT*	9221	9285	65				4	L
*trnS2*	9295	9359	65				0	H
*Cytb*	9358	10,497	1140	379	ATG	TAA	3	H
*ND6*	10,490	11,002	513	170	ATG	TAG	−2	H
*trnP*	11,004	11,068	65				0	H
*ND1*	11,073	12,014	942	313	ATG	TAA	1	H
*trnL2*	12,015	12,079	65				−40	H
*trnL1*	12,080	12,143	64				−31	H
*16S*	12,104	13,500	1397				2	H
*trnV*	13,470	13,536	67				9	H
*12S*	13,546	14,460	915				23	H
*trnM*	14,459	14,527	69				−2	H
*trnC*	14,536	14,599	64				8	H
*trnY*	14,600	14,663	64				0	H
*trnW*	14,664	14,734	71				0	H
*trnQ*	14,735	14,802	68				0	H
*trnG*	14,803	14,866	64				0	H
*trnE*	14,867	14,934	68				0	H

**Table 3:** The gene annotations of the complete mitogenomes of *A. pelagicus*.

Gene	Position		Size (bp)		Codon		Intergenic Nucleotides	Strand
	From	To	Nucleotide	Amino Acid	Initiation	Stop		
*COIII*	10	807	798	266	ATA	TAA	0	L
*trnK*	813	881	69				5	L
*trnA*	882	945	64				0	L
*trnR*	947	1008	62				1	L
*trnN*	1009	1076	68				0	L
*trnI*	1077	1142	66				0	L
*ND3*	1143	1439	351	117	ATG	TAA	0	L
*TrnS1*	1492	1560	69				−2	L
*ND2*	1561	2601	1041	347	ATG	TAA	0	L
*COI*	2573	4105	1533	511	ATG	TAA	−29	L
*COII*	4107	4793	687	229	ATG	TAA	1	L
*trnD*	4792	4855	64				−2	L
*ATP8*	4856	5011	156	52	ATG	TAA	0	L
*ATP6*	5016	5705	690	230	ATA	TAG	4	L
*trnF*	5731	5795	65				25	H
*ND5*	5796	7482	1687	562	ATG	T--	0	H
*trnH*	7483	7546	64				0	H
*ND1*	7564	8508	945	315	ATA	TAG	17	H
*trnL2*	8506	8570	65				−3	H
*trnC*	8597	8661	65				26	H
*trnY*	8662	8724	63				0	H
*trnE*	8734	8797	64				9	H
*ND4*	9907	11,241	1335	445	ATT	TAG	0	H
*ND4L*	11,247	11,543	297	99	ATG	TAG	5	H
*trnT*	11,548	11,612	65				4	L
*trnS2*	11,613	11,676	64				0	H
*Cytb*	11,675	12,814	1140	380	ATG	TAA	−2	H
*ND6*	12,807	13,319	513	171	ATG	TAG	−8	H
*trnP*	13,321	13,385	65				1	H
*trnL1*	13,598	13,665	68				0	H
*16S*	13,631	15,028	1398				−35	H
*trnV*	15,000	15,067	68				−29	H
*12S*	15,080	16,025	946				12	H
*trnM*	16,029	16,094	66				3	H
*trnW*	16,131	16,196	66				36	H
*trnQ*	16,197	16,264	68				0	H
*trnG*	16,267	16,336	70				2	H

**Table 4:** The nucleotide composition in the mitochondrial genome.

Species		Size (bp)	A%	T%	C%	G%	A + T%	C + G%	A-T Skew	G-C Skew
*J. diaphana*	Whole genome	16,111	40.6%	36.3%	15.4%	7.7%	76.9%	23.1%	0.056	−0.333
	13 PCGs	11,152	31.9%	43.5%	11.0%	13.5%	75.4%	24.5%	−0.154	0.102
	rRNA	2312	37.1%	42.5%	6.0%	14.4%	79.6%	20.4%	−0.068	0.412
	tRNA	1462	37.9%	40.6%	8.1%	13.4%	78.5%	21.5%	−0.034	0.247
*A. pelagicus*	Whole genome	17,380	40.6%	35.1%	16.8%	7.5%	75.7%	24.3%	0.073	−0.383
	13 PCGs	11,173	30.7%	42.7%	12.2%	14.4%	73.4%	26.6%	−0.163	0.083
	rRNA	2344	36.9%	41.6%	6.1%	15.4%	78.5%	21.5%	−0.060	0.433
	tRNA	1448	38.4%	40.1%	8.0%	13.5%	78.5%	21.5%	−0.022	0.256

## Data Availability

The annotated mitogenomes of *Japetella diaphana* and *Amphitretus pelagicus* were submitted and are available in the NCBI nucleotide database under accession numbers ON060363 and ON060364, respectively.
